# Notch It Up: Nudging Stem Cells toward a Neural Fate

**DOI:** 10.1371/journal.pbio.0040146

**Published:** 2006-04-11

**Authors:** Liza Gross

From the black widow spider to the six-toed sloth, every multicellular organism starts life as a single cell. This cell and the embryonic stem cells it spawns will live or die, grow, proliferate, migrate, and differentiate at the direction of a tightly controlled genetic program. Embryonic stem cells can self-renew to produce populations of identical cells that retain the ability to turn into any cell type of the body, a feature called pluripotency. Identifying the molecular signals that govern the maintenance and release of the pluripotent state would help scientists refine their ability to use embryonic stem cells as models of disease, as test beds for drug screening, and as a source for cell-based therapies. Researchers are particularly interested in uncovering the early signals that commit a cell to a particular fate, such as a skin, gut, or nerve cell.

Cell fate is determined in a wide variety of vertebrates and invertebrates by proteins called Notch receptors, which straddle the membrane of cells and transmit signals through local cell interactions. Depending on the context, Notch signaling can inhibit the spread of differentiation among adjacent cells or prompt them to adopt similar fates. In a new study, Sally Lowell, Austin Smith, and their colleagues discovered that Notch signaling also induces embryonic stem cells to make the initial commitment to a nervous system fate.

Working with undifferentiated mouse embryonic stem cells, Lowell et al. first confirmed that the cells express both the Notch receptor and its activators, or ligands. When the Notch pathway is activated, the receptor's intracellular domain (NotchIC) detaches and enters the nucleus. Once inside the nucleus, NotchIC binds to and activates the RBPJκ transcription factor, which in turn activates target genes. To boost Notch signaling without having to depend on complex ligands, the researchers engineered a transgene to allow ongoing, moderate expression of active NotchIC. Cells that showed this constitutive NotchIC expression, termed R26NotchIC cells, served as the experimental model. These cells come from an embryonic stem cell line (called 46C) that has green fluorescent protein linked to a neural specification marker gene *(Sox1)*, making it easy to identify cells that choose a neural fate.

With a suitable experimental system in hand, Lowell et al. released R26NotchIC cells and a control cell line from the influence of self-renewal factors in order to allow differentiation. By the second day, they saw a roughly 3-fold increase in glowing *Sox1*-expressing cells compared with the control line. The researchers also observed reduced levels of a key marker of pluripotency (Oct4) and sharp increases of a protein associated with the initial stages of differentiation (FGF5). These findings, along with the observation that R26NotchIC cells give rise to a coherent mass of glowing *Sox1* cells, indicate that NotchIC accelerates the onset of neural differentiation. The researchers go on to show that Notch not only guides cells into a neural fate—amplifying and coordinating induction within a cell population—but also restricts them from choosing other fates.

To investigate whether NotchIC is necessary for neural induction, the researchers interfered with Notch activity. When 46C cells were treated with an agent that blocks Notch activation by preventing cleavage of its intracellular domain, the number of cells that activated the *Sox1* neural marker was reduced to only 10% of normal, and most retained expression of the Oct4 pluripotency marker. Eventually, the cells differentiated, but not into neural cells. When this inhibitor was used to treat R26NotchIC cells, which have the already-cleaved receptor, *Sox1* expressed was unaffected; thus, the anti-neural effects come specifically from blocking Notch signaling. Using embryonic stem cell lines without functional *RBPJκ* genes (needed to activate Notch's target genes) produced similar results: the cells yielded far fewer *Sox1* cells and either retained Oct4 or differentiated into a non-neural cell type.

Finally, Lowell et al. tested Notch's effects in human embryonic stem cells and show that it works much like it does in mouse stem cells, guiding them toward a neural fate. By revealing an unexpected role for Notch in directing early differentiation, the researchers have identified a key molecular determinant of stem cell regulation. As scientists identify more and more of these critical molecular cues, the closer they will come to harnessing the power and promise of these much-embattled, protean cells.

## 

**Figure:**
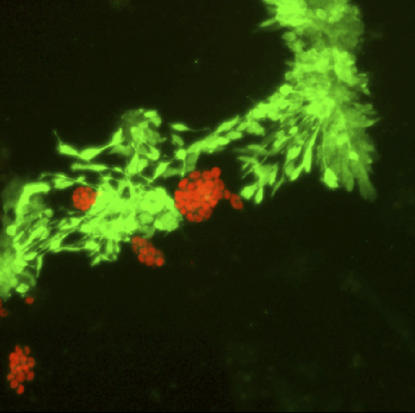
Neural progenitors (green) were efficiently generated from embryonic stem cells (red) through activation of the Notch pathway.

